# PLAC8 as a potential therapeutic target for myocardial infarction: unraveling the molecular mechanisms

**DOI:** 10.1007/s13105-025-01098-3

**Published:** 2025-06-05

**Authors:** Yifan Tong, Xin Huang, Wei Qian, Lijuan Liu

**Affiliations:** 1 Department of Cardiology, The First Affiliated Hospital, Jiangxi Medical College, Nanchang University, 17 Yongwai Zhengjie, Nanchang, Jiangxi Province 330006 China; 2 Department of Cardiology, The Third Affiliated Hospital, Jiangxi Medical College, Nanchang University, Nanchang, Jiangxi Province China

**Keywords:** Myocardial infarction, PLAC8, Hypoxia, Apoptosis, MEK/ERK

## Abstract

The incidence of myocardial infarction (MI) has been increasing in recent years, and the cause of acute myocardial infarction is apoptosis due to insufficient coronary myocardial blood supply. PLAC8 is a critical gene in the disease process of MI through GEO database research and analysis of differentially expressed genes (DEGs). In this study, in mice with myocardial infarction caused by surgical ligation of the left anterior descending coronary artery (LAD) and hypoxia-induced H9C2 cells as a model, the myocardium of the model group was found to show severe cardiomyocyte disorders, apoptosis of inflammatory cell infiltration, and ischemic state by HE, TTC, and Tunel staining. The expression of PLAC8 was reduced in the disease model by PCR and Western blot, and the expression of cle-Casp3 and Bax was also found to be high. However, overexpression of PLAC8 in the disease model reversed these processes. MEK/ERK and P65 are the core signaling pathways in the MI model. In this study, we found that the therapeutic effect of PLAC8 was related to the inhibition of the MEK/ERK signaling pathway by overexpression of PLAC8 and antagonism of the MEK/ERK signaling pathway. In conclusion, the inhibition of the MEK/ERK signaling pathway by PLAC8 under hypoxic conditions reduces apoptosis in H9c2 cells, which may provide new ideas for the determination and treatment of MI.

## Introduction

Myocardial infarction (MI) is a pathological process of ischemia and necrosis of the myocardium due to obstruction of the coronary arteries caused by rupture of atherosclerotic plaques in the coronary arteries, platelet aggregation, and clot formation. It usually occurs when myocardial tissue does not receive sufficient oxygen and nutrients due to inadequate blood supply to the coronary arteries [1, 2]. Acute Myocardial Infarction With the introduction of early reperfusion therapy, these complications now occur in less than 0.1% of patients after acute myocardial infarction. However, there has not been a parallel decline in mortality [3]. Cardiogenic shock occurs immediately after acute myocardial infarction in up to 10% of patients, with a mortality rate approaching 40% within 30 days and 50% within 1 year [4]. Myocardial infarction leads to loss of cardiomyocytes, subsequent pathological remodeling of the ventricles, and dramatic impairment of cardiac function, ultimately leading to heart failure. A growing number of studies have shown that the proliferative capacity of adult mammalian cardiomyocytes is very limited and insufficient to restore the injured heart. Therefore, finding a safe and effective therapeutic strategy against cardiomyocyte apoptosis is important to achieve cardiac repair after myocardial infarction [5, 6].

Placenta-specific 8 (PLAC8) was first identified during genome-wide expression profiling of mid-gestation placentas and embryos using 15,000 mouse developmental cDNA microarrays [7, 8]. To date, PLAC8 has been identified as being involved in organ development and tumourigenesis [9–11]. In addition, PLAC8 is a molecular marker for predicting prognosis and distinguishing between different cell subpopulations [12]. The current study found that the PLAC8 protein does not have an N-terminal signal peptide, suggesting that the protein is not a secreted protein but functions in the cytoplasm or nucleus. The intracellular distribution of the PLAC8 protein is dynamic and regulated in an implantation-dependent manner [13].

The MEK/ERK signaling pathway is a classical cellular proliferation and survival pathway that regulates cell proliferation, survival, and transcriptional activity by phosphorylating a series of downstream proteins [14]. However, excessive MEK/ERK signaling pathway activation in the MI can lead to myocardial fibrosis and remodeling. After MI, injured cardiomyocytes release inflammatory factors and extracellular matrix proteins, activating downstream molecules of the MEK/ERK signaling pathway and promoting myocardial fibrosis and scar tissue formation. This fibrotic process further deteriorates cardiac function [15, 16]. It was found that inhibition of the ERK signaling pathway in MI rats improved the microenvironment after myocardial infarction, thereby promoting the survival and efficacy of the implanted MSCs [17]. Therefore, regulating the MEK/ERK signaling pathway may be a potential therapeutic target for treating myocardial infarction.

In the present study, we were interested in the role of PLAC8 in myocardial infarction-induced myocardial injury and its intrinsic mechanism. However, current reports on the relationship between PLAC8 and the MEK/ERK pathway and with MI-induced myocardial injury are unclear.

## Materials and methods

### Microarray data retrieval

The keyword “myocardial ischemia” was used as a keyword to screen in the Gene Expression Omnibus (GEO) database. We further filtered based on the information of sequencing type (transcriptology), animal species (mouse), sample source (ventricle), and modeling time. Finally, we obtained GSE161151. which was generated by the GPL20775 platform and contains 3 mouse left ventricular tissue samples. Each LV tissue was divided into three regions: normal zone, ischemia zone and ischemia border zone. In order to better analyze the differential genes between the MI group and the control group (Sham), we selected three pairs of the normal zone and ischemia zone paired samples for analysis.

### Acquisition of microarray data and identification of differentially expressed genes (DEGs)

Data from GSE161151 were downloaded and read from GEO using the R package “GEO query.” The DEGs of each microarray were analyzed using the GEO2R online tool, where the parameters were set to *p* < 0.05 and|log2 (Fold-change)| ≥ 0.25. The volcano Plot and Heatmap were plotted using the R package “ggplot2” and the R package “ComplexHeatmap,” respectively. PLAC8 was among the significantly downregulated DEGs in the ischemic zone compared to the normal zone, as determined by GEO2R analysis under defined statistical criteria.

### Animals

All 6–8 week-old male C57BL/6 J mice were purchased from Shanghai Slac Co. and housed in the SPF Laboratory Animal Centre. The mice were kept in their new environment for at least one week before formal experiments. During the whole experiment, the mice will live in an environment with a temperature of 24 ± 2℃, a relative humidity of 60 ± 5%, and an air cleanliness of class 7. The mice will be exposed to 12 h of light and 12 h of darkness per day and are guaranteed adequate food and acidified water. Every effort was made to minimize animal suffering. At the end of the experiment, mice were euthanized with 90% CO2. All animals received humane care, and experimental procedures were performed in accordance with the guidelines of the Nanchang University for the health and care of experimental animals. The protocol was reviewed and approved by the Experimental Animal Ethical Committee of the Nanchang University.

### MI surgery and PLAC8 upregulation

All mice were anesthetized in chambers with 2% isoflurane under sterile conditions. Myocardial infarction was caused by ligation of the left anterior descending coronary artery (LAD) with a 7 − 0 silk thread, the chest was opened from the left fourth intercostal space, and the LAD was sutured with a 6 − 0 silk thread 1 mm below the apical portion of the left atrial appendage. Sham-operated mice underwent the same surgical procedure, but the left anterior descending coronary artery was not bound. Successful induction of myocardial infarction was verified by observing myocardial blanching and ischemic discoloration.

In animal experiments, genepharma (Shanghai, China) constructed adeno-associated virus 9 (Ad-PLAC8) and control virus (Ad-NC) carrying PLAC8. Mice were injected via the tail vein to overexpress PLAC8 at a viral load of 1.1*1011 per mouse. In cellular experiments, PLAC8-carrying adenoviral vector (PLAC8-OE) and control virus (empty adenoviral vector [vector]) were infected into H9C2 cells to control the expression of PLAC8. Inclusion of both sham and vector controls ensured that the observed effects were specific to PLAC8 overexpression.

### H9C2 cell line and Hypoxia-induced conditions

H9C2 cells were purchased from the ATCC and grown in a DMEM medium containing 10% FBS and 1% penicillin-streptomycin. The environment for cell survival was maintained at 37 °C, 5% CO2, and suitable humidity.

Under cellular hypoxia, this research changed the survival environment of H9c2 to 93% N2, 2% O2, and 5% CO2 induced for 24 h, and the rest of the conditions were kept unchanged. The control group maintained its original survival status.

For U0126 pretreatment, U0126 was dissolved in dimethyl sulfoxide (DMSO). For cardiomyocytes, an aliquot of U0126 stock solution was added to the medium before hypoxia to ensure that the final U0126 working solution concentration was 50 µmol/L and that DMSO was < 0.01%. Equal concentrations of DMSO were added to the control group.

### qPCR

All myocardial tissue samples or H9C2 cells were homogenized for RNA isolation using the TRIZOL method. After determining RNA purity (1.8-2.0), it was reverse transcribed and subjected to the PCR reaction. The PCR process includes reverse transcription, pre-metamorphosis, a PCR cycling step and a complementary extension step. Among the RNA primers was PLAC8, and β-actin was used as a reference.

### Western-blot

All samples were transferred to RIPA buffer at low temperature after homogenization and soaked for 30 min. Pure protein samples were isolated by high-speed centrifugation (12000 rpm, 4 °C, 10 min). Protein samples were quantified under a BCA kit and had SDS-PAGE gel electrophoresis for separation. Images were developed and retained after incubation with appropriate antibodies. The primary antibodies included PLAC8, ANP, BNP, CTNT, CTNI, p-MEK, MEK, p-ERK, ERK, p-p65, p65, cle-Caspase3, BAX. β-actin was used as an internal reference for the total proteins, and lamin B was used as an internal reference for the nucleus.

The nuclear protein extraction method for all samples was performed in full accordance with the Nuclear and Cytoplasmic Protein Extraction Kit.

### HE staining

At the end of the experiment, myocardial samples were washed in pre-cooled PBS buffer, fixed in 4% paraformaldehyde, dehydrated in gradient ethanol and xylene, and embedded in paraffin. The lesion area was selected and serially sectioned, and the sections were photographed under a bright-field microscope after hematoxylin and eosin staining.

### TTC staining

Infarct area was determined by triphenyltetrazolium chloride (TTC) staining [18, 19]. Briefly, at the end of the experimental protocol, mouse hearts were perfused with 1% TTC for 10 min and snap-frozen. Subsequently, sections were made along the long axis of the heart and rinsed in 10% formaldehyde solution to remove color, as well as digitally photographed for planimetry. Infarct area was expressed as the ratio of the infarct zone to the danger zone (in this model of global ischemia, the danger zone is the entire ventricular volume).

### Tunel staining

Sections containing myocardial tissue were deparaffinized and incubated with a drop of 20 µg/ml proteinase K for 15–30 min at 37 °C, during which time the sections were kept humid. The sections were washed 3 times/5 minutes with PBS to remove excess proteinase K. The sections were incubated with TUNEL solution for 60 min at 37 °C, protected from light. The sections were washed 3 times/5 minutes with PBS, added DAPI working solution for 10 min and rinsed under running water for 5 min to seal the sections with sealing gel. Photographs were taken under a fluorescence microscope.

### Annexin V/PI

The treated cells were digested with trypsin and transferred to a 1.5 mL tube, washed three times with PBS to remove excess trypsin. Cells were resuspended with 195 µl of binding buffer, as well as 5 µL of Annexin V-FITC and 10 µL of PI-PE staining solution. Cells were incubated in the dark for 20 min at room temperature, and fluorescence was captured by the FITC and PE channels of the flow cytometer.

### PI staining

The treated cells were washed three times with PBS, and 10 µL of PI-PE staining solution was added. The cells were incubated in the dark at room temperature for 20 min, washed three times with PBS, and then the slices were sealed with DAPI-containing glycerol. The fluorescence was collected by fluorescence microscope.

### Elisa

All serum handling, dilutions, and procedures are subject to the requirements of the ELisa reagent vendor.

### Statistical analysis

The significant differences between groups were evaluated using one-way Analysis of Variance (ANOVA) using SPSS 25.0. For post hoc analysis, we used Tukeys post hoc tests. Statistical significance was determined at *P* < 0.05, with results expressed as mean ± standard deviation.

## Results

### PLAC8 levels are significantly inhibited in myocardial ischemia and hypoxia-induced H9C2

**Fig. 1:**
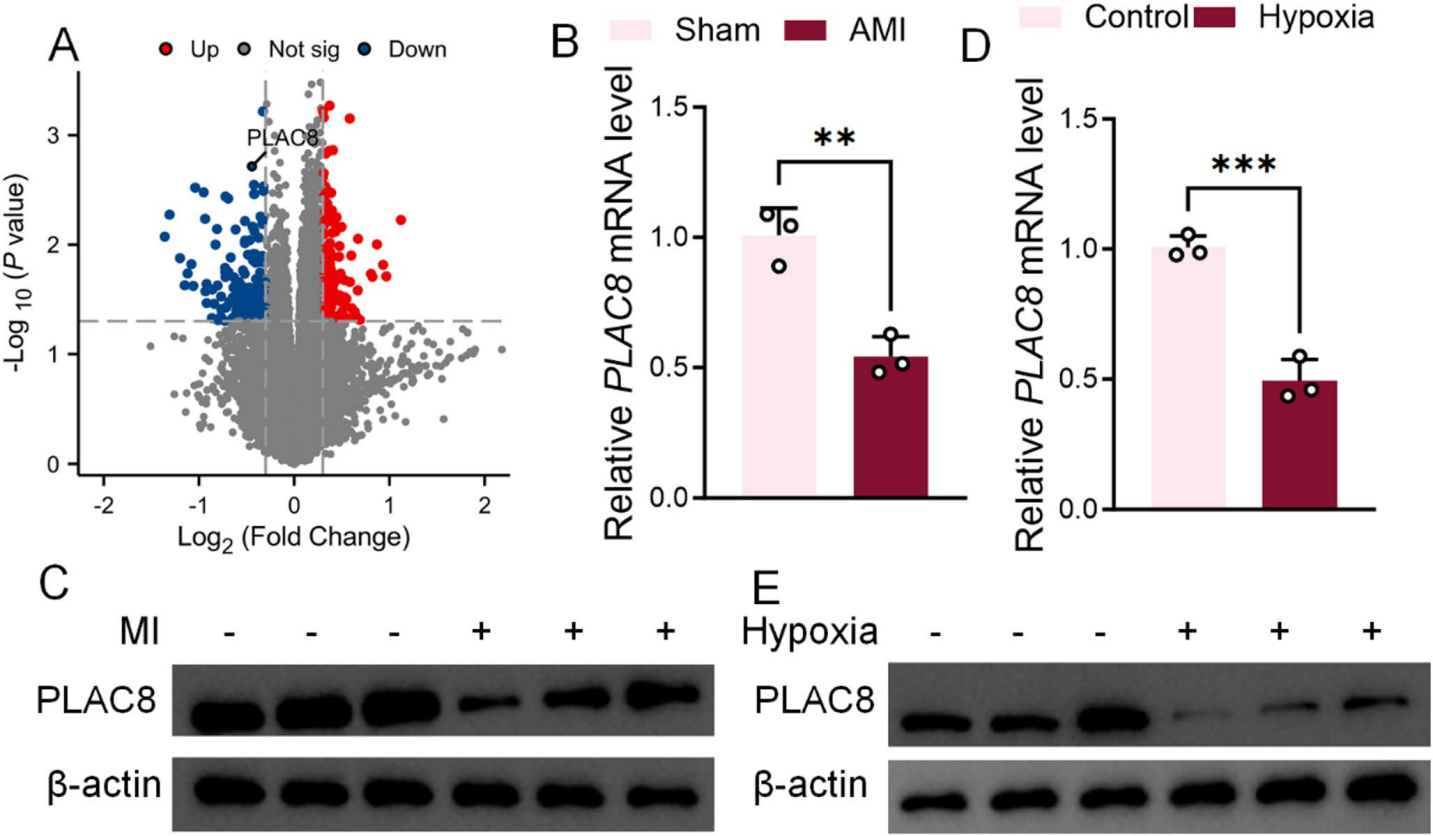
PLAC8 levels are significantly inhibited in myocardial ischemia and hypoxia-induced H9C2. (**A**) Volcanogram analysis of postoperative differentially regulated genes in permanently occluded anterior interventricular arteries; (**B**) mRNA levels of myocardial PLAC8 in MI mice were detected by PCR; (**C**) Western Blot examined protein levels of myocardial PLAC8 in MI mice; (**D**) mRNA levels of PLAC8 in hypoxia-induced H9C2 were examined by PCR; (**E**) protein levels of PLAC8 in hypoxia-induced H9C2 were examined by PCR

With the assistance of the GEO2R analysis tool, PLAC8 showed significantly lower expression in tissues with myocardial ischemia, as screened by expression profiling of the GSE161151 database (Fig. 1A). In the present study, PLAC8 mRNA and protein levels were significantly decreased in MI tissues compared with Sham controls and in hypoxia-induced H9c2 cells compared to controls. (Fig. 1B, C). Similar results were seen in hypoxia-induced H9C2 cells, which showed lower PLAC8 gene and protein expression than control H9C2 cells (Fig. 1D, E).

### Mice overexpressing PLAC8 show lower myocardial injury after MI surgery

**Fig. 2:**
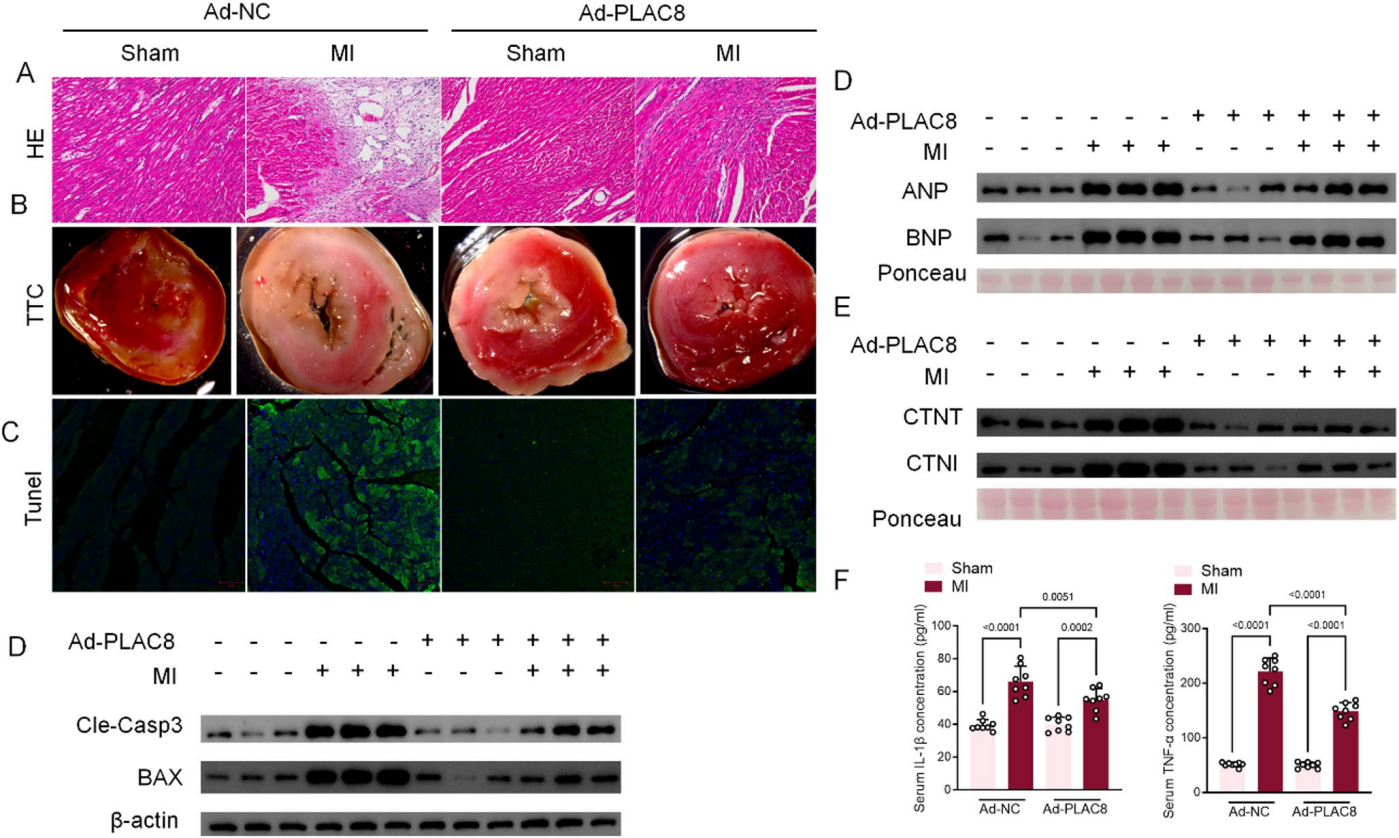
Mice Overexpressing PLAC8 Show Lower Myocardial Injury After MI Surgery. (**A**) Myocardial morphological staining was done by HE staining; (**B**) Myocardial ischaemic status was reflected by TTC staining.; (**C**) myocardial apoptosis in AMI mice was detected by Tunel staining; (**D**) Cle-Casp3 and BAX in myocardial tissue were detected by Western Blot assay. (**E**, **F**) ANP, BNP, CTNT, and CTNI in the serum of mice were detected by Western Blot, and (**G**) IL-1β and TNF-α in the serum of mice were detected by ELISA

Compared with Ad-NC-sham mice, mice in the Ad-NC-MI group exhibited a disordered myocardial tissue arrangement order and a large infiltration of inflammatory cells (Fig. 2A), while the myocardium of the mice showed significant ischemia, an enlarged infarct area (Fig. 2B), and significant apoptosis of cardiomyocytes (Fig. 2C). Compared with Ad-NC-MI mice, mice in the Ad-PLAC8-MI group presented with improved myocardial tissue alignment and a significant reduction in inflammatory cell infiltration (Fig. 2A), as well as controlled myocardial ischemia, reduced infarct area (Fig. 2B), and reduced apoptosis of cardiomyocytes (Fig. 2C). Importantly, the myocardium of mice in the Ad-PLAC8-Sham group, on the other hand, was similar to that of mice in the Ad-NC-Sham group, maintaining a relatively healthy state. ANP, BNP, CTNT, and CTNI are sensitive markers commonly used to indicate the status of cardiomyocytes, and a high expression of these markers is indicative of cardiomyocyte damage. A similar situation was found by protein level assay, in which the serum levels of Cle-Casp3 and BAX proteins in Ad-PLAC8-MI mice showed a significant reduction compared with those in Ad-NC-MI mice, indicating less apoptosis in myocardial tissues. ANP, BNP, CTNT, and CTNI are sensitive markers commonly used to indicate the status of cardiomyocytes, and high expression of these markers is indicative of cardiomyocyte damage. Compared to Ad-NC-sham mice, Ad-NC-MI mice have abnormally elevated serum levels of ANP, BNP, CTNT, and CTNI proteins, indicating severe myocardial tissue damage and risk of heart failure. Compared with Ad-NC-MI mice, Ad-PLAC8-MI mice showed significantly reduced serum levels of ANP, BNP, CTNT, and CTNI proteins, suggesting that myocardial tissue damage was ameliorated to some extent. Consistent results were also obtained in serum ELISA (Fig. 2E, F). Compared with Ad-NC-MI mice, Ad-PLAC8-MI mice showed significantly reduced serum levels of inflammatory indicators IL-1β and TNF-α, suggesting an ameliorative effect (Fig. 2G).

### MI mice with overexpression of PLAC8 inhibit the MEK/ERK pathway as well as the NFKB pathway in myocardial tissue

**Fig. 3:**
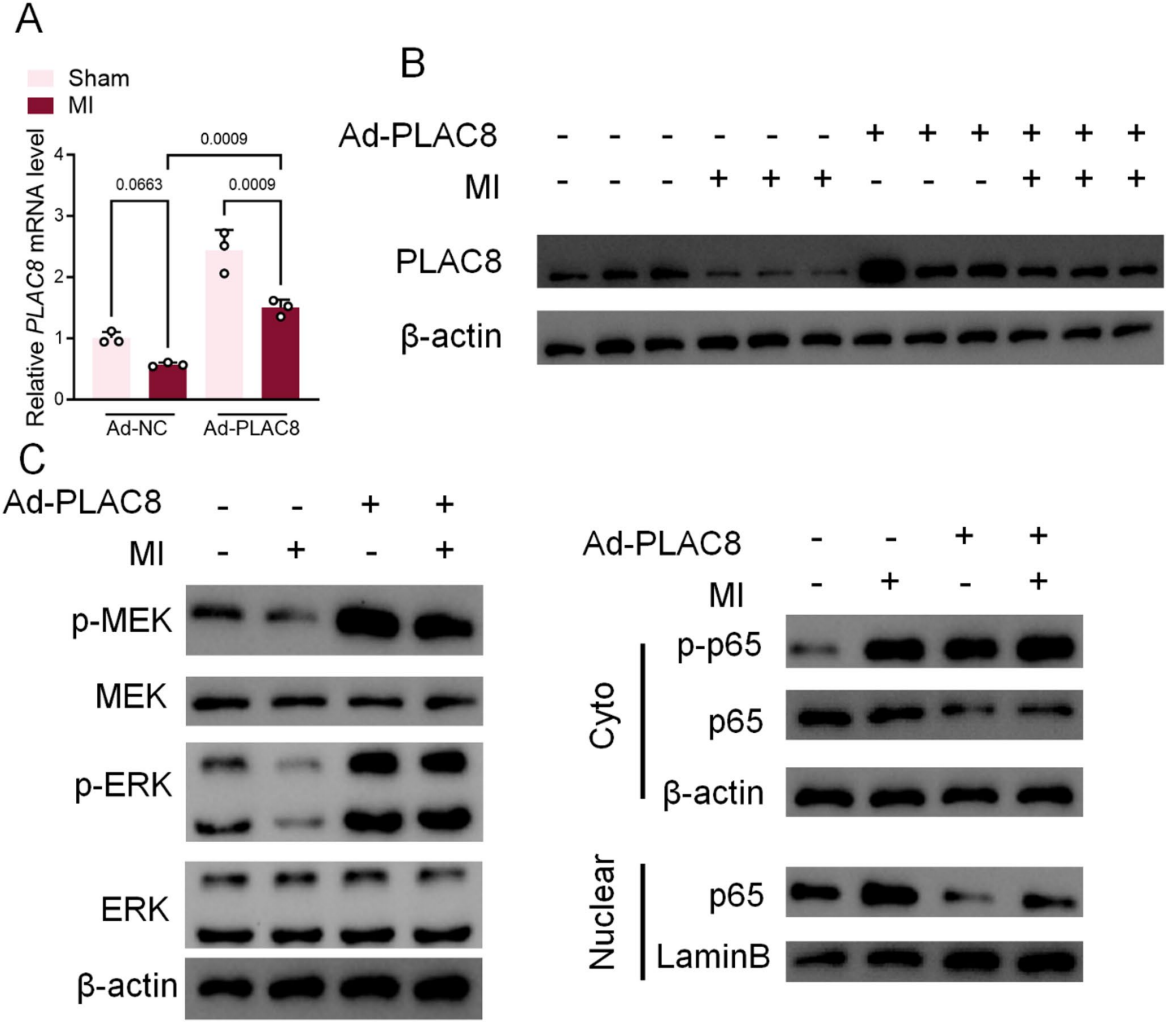
MI mice with overexpression of PLAC8 inhibit the MEK/ERK pathway as well as the NFKB pathway in myocardial tissue. (**A**) PLAC8 mRNA in myocardial tissue was detected by qPCR. (**B**, **C**) PLAC8, p-MEK, MEK, p-ERK, ERK, p-p65, p65 in myocardial tissue or nuclei were detected by Western Blot

The present study found a correlation between the state of the myocardium and the expression of PLAC8. Higher PLAC8 gene and protein expression was seen in the myocardium of Ad-PLAC8-MI mice compared with Ad-NC-MI mice (Fig. 3A, B). The MEK/ERK pathway, as well as the NFKB pathway appearing highly expressed in myocardial infarction, has been uncovered [20, 21]. Western blot and nuclear fractionation confirmed the activation of MEK/ERK and NF-κB/p65 pathways in MI tissues. Myocardial tissues of Ad-PLAC8-MI mice showed significantly decreased protein levels of p-MEK/MEK and p-ERK/ERK compared with Ad-NC-MI mice. Similarly, p65, a key core protein in NFKB, showed similar results. p-p65/p65 protein levels were significantly decreased in myocardial tissues of Ad-PLAC8-MI mice compared with Ad-NC-MI mice. Isolation of nuclei from fresh myocardial tissue cells revealed that the fraction of p65 protein entering the nucleus was significantly downregulated in the myocardial tissue of Ad-PLAC8-MI mice (Fig. 3C).

### Overexpression of PLAC8 restores hypoxia-induced H9C2 damage and is associated with Inhibition of the MEK/ERK pathway and the NFKB pathway

**Fig. 4:**
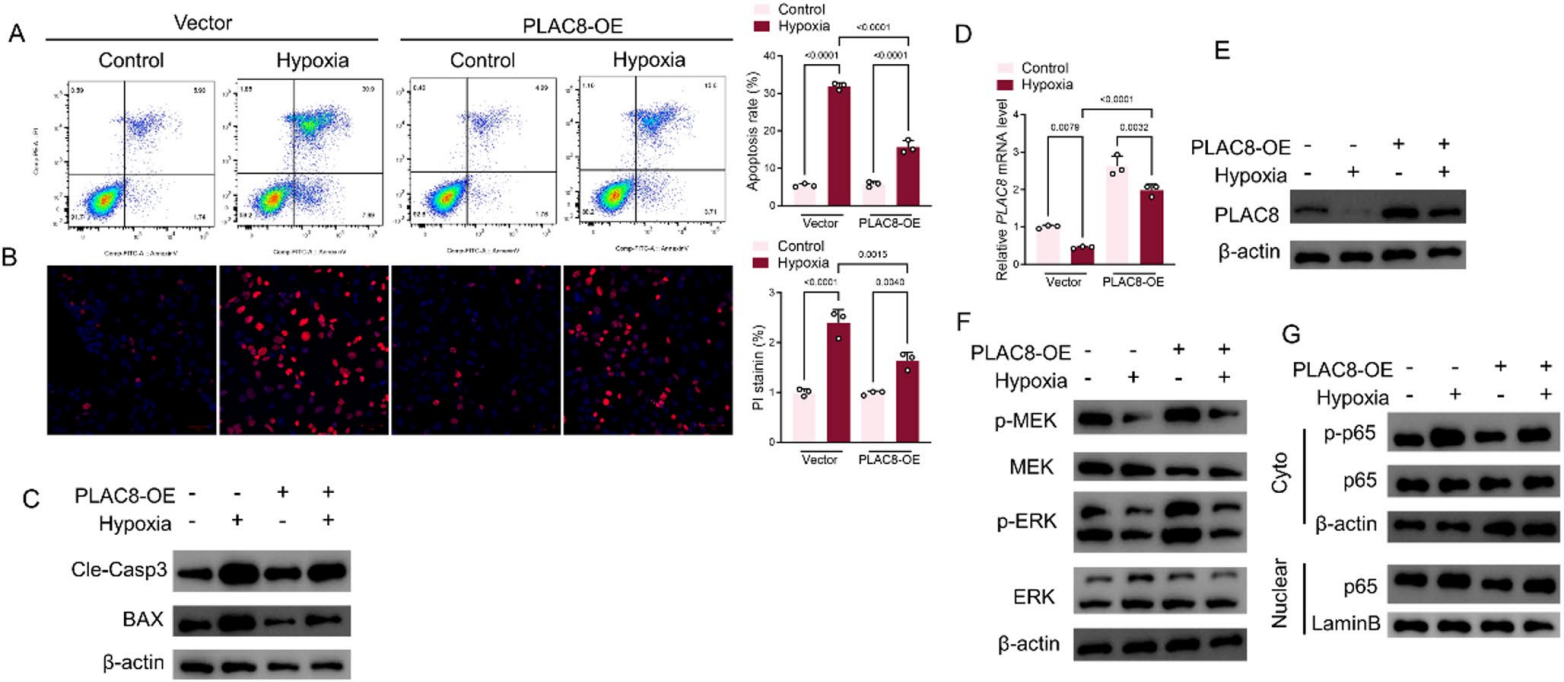
Overexpression of PLAC8 restores hypoxia-induced H9C2 damage and is associated with inhibition of the MEK/ERK pathway and the NFKB pathway. (**A**) Annexin V/PI detection of H9C2 cell apoptosis; (**B**) PI staining to detect H9C2 cell apoptosis; (**C**) H9C2 cell PLAC8 protein was detected by Western Blot assay. (**D**) H9C2 cell PLAC8 mRNA was detected by PCR assay; (**E**, **F**) H9C2 cell PLAC8, p-MEK/MEK, p-ERK/ERK protein was detected by Western Blot assay. (**G**) The p65 protein in H9C2 cells or nuclei was detected by Western blot

Through the simulated environment of hypoxia, H9C2 cells showed a markedly elevated apoptosis rate significantly, an increase in PI fluorescence intensity, as well as an increase in the expression of the core indicators of apoptosis, Cle-Casp3, and BAX proteins. However, in cells overexpressing PLAC8, H9C2 cells challenged with hypoxia showed a marked regression of apoptosis rate, a decrease in PI fluorescence intensity, and a decrease in the expression of Cle-Casp3, a core indicator of apoptosis, and BAX proteins (Fig. 4A-C). The same occurred in the MEK/ERK and NFKB signaling pathways. In overexpression. Compared with H9C2 cells in the Vector-Hypoxia group, PLAC8 mRNA and protein levels in H9C2 cells in the PLAC8-OE-Hypoxia group appeared to be significantly elevated (Fig. 4D, E). In contrast, the protein expression of p-MEK/MEK, p-ERK/ERK, and p-p65/p65 appeared significantly downregulated (Fig. 4F, G). The nuclei of H9C2 cells were isolated and detected separately, and it was found that the expression level of p65 was significantly decreased in the nuclei of H9C2 cells in the PLAC8-OE-Hypoxia group (Fig. 4G).

### MEK inhibitor U0126 reverses the therapeutic effects of overexpressed PLAC8

**Fig. 5:**
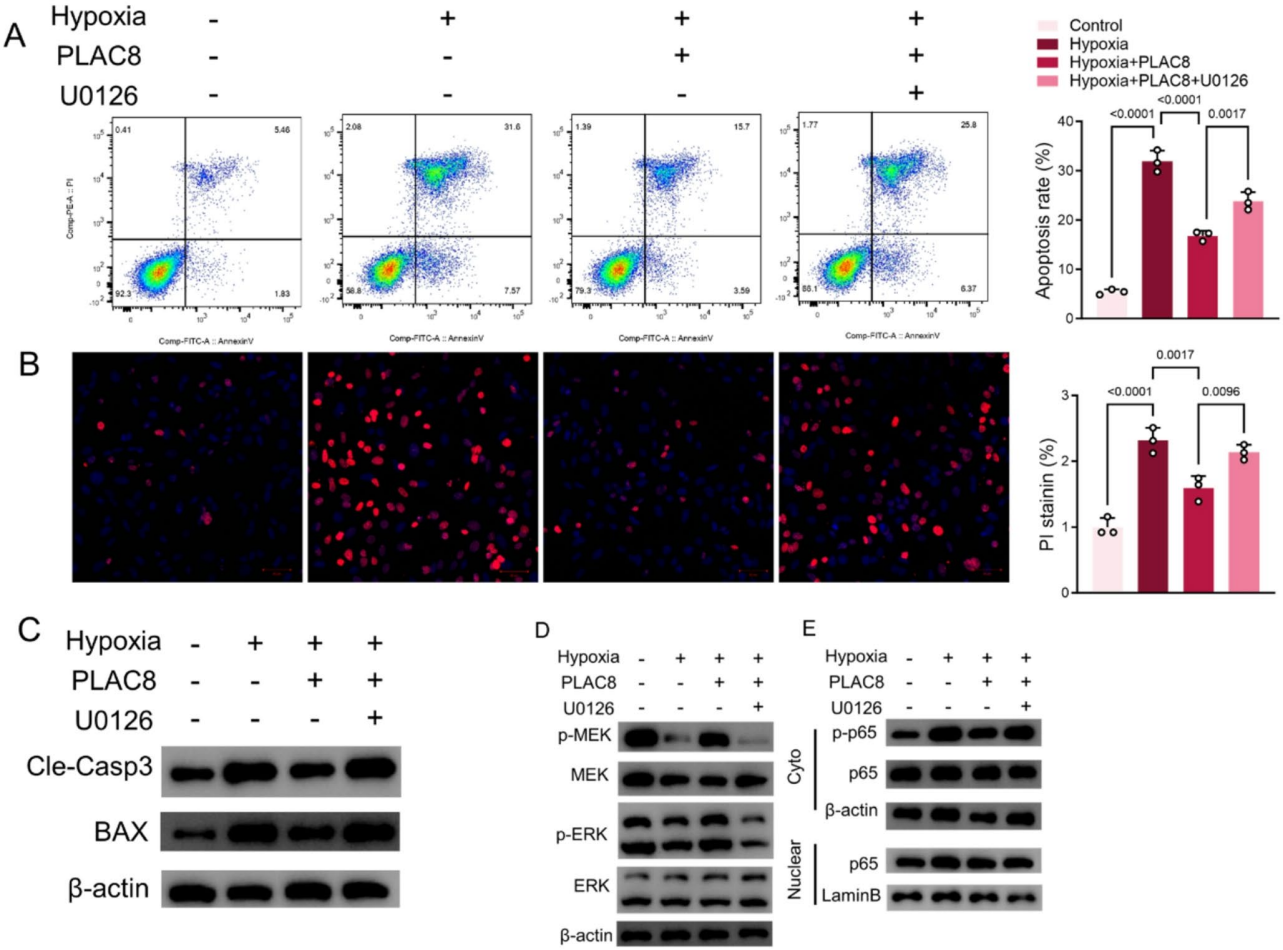
MEK inhibitor U0126 reverses the therapeutic effects of overexpressed PLAC8. (**A**) Annexin V/PI detection of H9C2 cell apoptosis; (**B**) PI staining to detect H9C2 cell apoptosis; (**C**) H9C2 cell Cle-Casp3, BAX protein was detected by Western Blot assay. (**D**) H9C2 cell p-MEK/MEK, p-ERK/ERK protein was detected by Western Blot assay. (**G**) The p65 protein in H9C2 cells or nuclei was detected by Western blot

The above findings have revealed that PLAC8 overexpression appeared to be protective under hypoxia and was associated with the MEK/ ERK signaling pathway.U0126, a MEK inhibitor, significantly increased the apoptosis rate, and PI fluorescence intensity of H9C2 cells overexpressing PLAC8 (Fig. 5A, B). Similar results were found at the Cle-Casp3 and BAX protein levels (Fig. 5C). Subsequently, at the protein level, U0126 reversed the inhibitory effects of p-MEK/MEK, p-ERK/ERK, p-p65/p65 and p65 entry induced by H9C2 upon overexpression of PLAC8 (Fig. 5D, E). Our data demonstrate that PLAC8 overexpression leads to a significant reduction in the phosphorylated forms of MEK and ERK, which coincides with reduced apoptotic markers and improved myocardial morphology. The reversal of these effects by U0126 further confirms the role of the MEK/ERK pathway in mediating the protective effects of PLAC8.

## Discussion

Myocardial infarction is one of the leading causes of death worldwide. While our study does not assess population-level trends, recent reports have demonstrated a concerning rise in MI among younger individuals, emphasizing the need for novel diagnostic and therapeutic approaches [22, 23]. During myocardial infarction, many cardiac cells die by various processes, such as apoptosis [24]. In recent years, searching for highly sensitive and specific marker molecules for early diagnosis, intervention and control of MI has become an important research hotspot regarding treatment and prognostic assessment [25].

The GEO database is a publicly available gene expression repository containing biological experimental data from various species and experimental designs. The GSE161151 database sample is an 8-week C57BL/6 mouse model of myocardial ischemia-reperfusion injury (45 min of ischemia and 24 h of reperfusion) found that the expression of PLAC8 in the myocardial infarction zone samples showed a significant downward trend.

Therefore, the present study similarly mimicked experiments in mice with myocardial infarction and hypoxia-induced H9C2 cells.

In this study, we established an in vitro cellular model of myocardial infarction by surgical ligation of the left anterior descending coronary artery (LAD) causing myocardial infarction in a mouse model and in hypoxia-treated cardiomyocytes H9c2. It was found that PLAC8 expression was significantly inhibited in both models, consistent with the results obtained from GEO analysis. Similarly, both myocardial-infarcted mice and hypoxia-inhibited H9c2 cells showed significant apoptosis. These results are consistent with those documented in the literature and indicate successful animal and cellular modeling [26]. It was also found that overexpression of the PLAC8 gene in MI mice and in hypoxia-challenged cardiomyocytes H9c2 significantly reduced Cle-Casp3 and BAX expression and apoptosis in cardiomyocytes. This evidence suggests a protective role of PLAC8 in cardiomyocytes.

Myocardial infarction is one of the common causes of heart failure. When a blockage in a coronary artery causes a myocardial infarction, a portion of the heart muscle tissue is damaged or dies. This leads to a decrease in the heart’s contractile function and a decrease in the elasticity of the heart muscle, which causes a reduction in the heart’s pumping function [27, 28]. Suppose the myocardial infarction affects a large area of the heart muscle or is not treated promptly. In that case, it may significantly impair the heart’s contractile function, which in turn may lead to heart failure. ANP, BNP, CTNT, and CTNI are common sensitivities in heart failure. In this study, it was found that overexpression of PLAC8 rescued the high expression of these markers, reversed the progression of myocardial infarction to heart failure, reduced cardiomyocyte injury, and lowered the expression of the inflammatory markers TNF-α and IL-1β in serum.

MEK/ERK and P65, as essential regulators of MI, have been reported in a large number of studies; for example, the MEK/ERK pathways are activated during cardiac hypertrophy after myocardial infarction in rats and oxygen-glucose deprivation/reperfusion (OGD/R) -induced H9c2 cells [29, 30].

P65 is a protein subunit of the NF-κB transcription factor complex. NF-κB is a crucial regulator of immune response and inflammation. P65 is one of the five subunits of NF-κB. It plays a vital role in activating the NF-κB signaling pathway [31, 32]. Similarly, blocking oxidative stress and nuclear translocation of P65 in mice after myocardial infarction significantly attenuated cardiac fibrosis and inflammation after myocardial infarction and acted as myocardial protection [33–35]. This study found that MI mice and hypoxia-induced H9C2 cells stimulate the activation of MEK/ERK and P65 signaling pathways due to low expression of PLAC8. In contrast, up-regulation of PLAC8 significantly inhibits the expression of MEK/ERK and P65. Based on these findings, pharmacological blockade of MEK was next performed (U0126) [36, 37], and we found that all the therapeutic effects induced by PLAC8 were reversed. This also reflects that PLAC8 regulates apoptosis in cardiomyocytes, mainly through the MEK/ERK signaling pathway.

## Conclusion

In summary, deficiency of PLAC8 activates the MEK/ERK signalling pathway to promote cardiomyocyte apoptosis in MI mice and hypoxia-induced H9c2 cardiomyocyte injury. This study provides theoretical support for the role of PLAC8 in MI.

## Data Availability

No datasets were generated or analysed during the current study.
